# Research transparency in dental research: A programmatic analysis

**DOI:** 10.1111/eos.12908

**Published:** 2022-12-08

**Authors:** Eero Raittio, Ahmad Sofi‐Mahmudi, Sergio E. Uribe

**Affiliations:** ^1^ Institute of Dentistry University of Eastern Finland Kuopio Finland; ^2^ Department of Dentistry and Oral Health Aarhus University Aarhus Denmark; ^3^ Seqiz Health Network Kurdistan University of Medical Sciences Seqiz Kurdistan Iran; ^4^ Department of Health Research Methods, Evidence and Impact McMaster University Hamilton Ontario Canada; ^5^ Department of Conservative Dentistry and Oral Health Riga Stradins University Riga Latvia; ^6^ School of Dentistry Universidad Austral de Chile Valdivia Chile; ^7^ Baltic Biomaterials Centre of Excellence Headquarters at Riga Technical University Riga Latvia

**Keywords:** clinical trial protocols, conflict of interest, dentistry, information dissemination, open access publishing

## Abstract

We assessed adherence to five transparency practices—data sharing, code sharing, conflict of interest disclosure, funding disclosure, and protocol registration—in articles in dental journals. We searched and exported the full text of all research articles from PubMed‐indexed dental journals available in the Europe PubMed Central database until the end of 2021. We programmatically assessed their adherence to the five transparency practices using a validated and automated tool. Journal‐ and article‐related information was retrieved from ScimagoJR and Journal Citation Reports. Of all 329,784 articles published in PubMed‐indexed dental journals, 10,659 (3.2%) were available to download. Of those, 77% included a conflict of interest disclosure, and 62% included a funding disclosure. Seven percent of the articles had a registered protocol. Data sharing (2.0%) and code sharing (0.1%) were rarer. Sixteen percent of articles did not adhere to any of the five transparency practices, 29% adhered to one, 48% adhered to two, 7.0% adhered to three, 0.3% adhered to four, and no article adhered to all five practices. Adherence to transparency practices increased over time; however, data and code sharing especially remained rare. Coordinated efforts involving all stakeholders are needed to change current transparency practices in dental research.

## INTRODUCTION

Open registration of protocols and their amendments, and access to data and the study report, are essential components of open science. When conducting and reporting research, transparency is essential for detecting or preventing research bias and also for assessing the credibility of scientific findings. The importance of research transparency for the proper evaluation of research findings using big data or involving artificial intelligence algorithms has also been emphasized [1]. In addition, some of these transparency practices include disclosure of funding, disclosure of conflicts of interest (COI), detailed reporting of methods that allow result replications, and publication of original data and code to ensure reproducibility and full use of the data [2, 3, 4].

A recent systematic review of almost 3 million biomedical studies showed major improvements in COI and funding disclosures but only minor improvements in protocol registration, data sharing, and code sharing since 2000 [4]. Smaller and less‐comprehensive studies have investigated transparency practices in dental research [5, 6]. They have shown far‐from‐optimal practices in funding and COI disclosures, protocol registration, and data sharing, which have been reported in a recent scoping review [5]. However, methodological differences and inconsistencies in definitions and reporting in primary studies did not allow the authors of that review [5] to obtain a comprehensive view of transparency practices in dental research. Consistent with the findings from the research articles, investigations of journal policies in dental journals have shown suboptimal adherence to transparency practices, for instance, by not requiring preregistration of trials [7, 8].

Monitoring transparency has been seen as an essential way to increase value and reduce waste in biomedical research [9] and should be of great interest to research funders [10, 11]. To the best of our knowledge, there are no reports of transparency indexes in the dental literature. For this, we assessed the adherence to five transparency practices—data sharing, code sharing, COI disclosure, funding disclosure, and statements of protocol registration—in open access full‐text articles published in dental journals available from the Europe PubMed Central (EPMC) database. We mapped transparency practices across publication years and according to journal and article‐related characteristics.

## MATERIAL AND METHODS

The protocol for this descriptive study is available on the Open Science Framework (OSF) (osf.io/h5p6a). All codes and data related to this study were shared via its OSF repository (osf.io/4eq8u) at the time of manuscript submission.

### Data sources and study selection

We searched for open‐access articles from dental journals available in the EPMC open science database. We reviewed dental journals based on a list of PubMed‐indexed dental journals provided by the National Library of Medicine catalogue (bit.ly/37XwnMt). We restricted our search to papers in English, and considered papers published until 31 December 2021, without a lower limit.

The search query for the EPMC database was as follows: ‘(ISSNs for all dental journals) AND (SRC:“MED”) AND (LANG:“eng” OR LANG:“en” OR LANG:“us”) AND (FIRST_PDATE:1900‐01‐01 TO 2021‐12‐31) AND (OPEN_ACCESS:y) AND (PUB_TYPE:“Journal Article” OR PUB_TYPE:“research‐article” OR PUB_TYPE:“rapid‐communication” OR PUB_TYPE:“product‐review”)’.

We downloaded all identified available records in XML full‐text format for full‐text evaluation using the *metareader* package [12], and the proportion of articles available as open access via the EPMC was calculated based on the total number of articles detected in the database.

### Data extraction and synthesis

We assessed adherence of articles to five transparency practices—COI disclosure, funding disclosure, protocol registration, data sharing, and code sharing—using a validated and automated tool (*rtransparent* package) developed by Serghiou et al. [4] to identify these five transparency practices programmatically (in an automated way). This tool identifies the five transparency practices based on phrases and their location in the article (e.g., the Acknowledgements section). For data sharing, code sharing, and protocol registration, the tool identifies whether the article had actually adhered to practice (for instance, the statement “data available on request” is not counted as sharing data), whereas COI and funding disclosure merely detect whether articles include the disclosure, regardless of its specific content [4]. For instance, COI disclosures are detected by identification of: (a) common phrases, such as “conflicts of interest” or “competing interests”; (b) common titles of sections with a COI disclosure (e.g., “Conflicts of Interests”); (c) common phrases in COI disclosures (e.g., “XX received commercial benefits from YY” or “No competing interests”); and/or (d) an acknowledgment section containing words or phrases common in COI disclosures (e.g., “fees”, “advisory board”, etc.). More information about the tool and its validation is described elsewhere [4].

For validation of the transparency practices identified in the sample articles, we manually checked the presence/absence of data sharing, code sharing, COI disclosure, funding disclosure, and protocol registration in 50 random articles from the sample using methods described by Serghiou et al. [4].

Basic journal‐ and article‐related information (publication year, citations of article, and journal name) was retrieved from the EPMC database. Publisher information was extracted from ScimagoJR (scimagojr.com), and journal impact factor (JIF) was obtained from the Journal Citation Reports (jcr.clarivate.com).

As indicated by earlier studies [4] and our preliminary investigations of this sample, information obtained from the EPMC database (from PubMed) on article type was inaccurate. Based on earlier literature [13], an algorithm was developed to classify the different types of studies using information from titles and abstracts. The algorithm was optimized in several rounds of tests and validation samples. In a final validation sample of 100 articles, the algorithm produced 78% agreement with manual categorization based on title and abstract (more information and code is available in the Supplementary Material of the OSF repository [osf.io/4eq8u]), which the authors deemed sufficient and provided guiding article‐type categorization.

### Data analysis

We used R v4.1.2 [14] for searches, data handling, analysis, and reporting. The searches and data export from EPMC were conducted using the *europepmc* package [15]. Indicators of transparency practices from the available full texts were extracted using the *rtransparent* package. Trends over time in transparency practices were reported in descriptive tabulations and graphical illustrations that were created using the *ggplot2* package [16]. As this type of programmatical (automated) detection is not perfect, we used the sensitivity and specificity of the *rtransparent* package [4] to generate 95% CIs for the prevalence estimates of the transparency practices we obtained using the *epiR* package [17]. We also calculated the number of transparency practices adhered to in articles, with that count ranging from 0 to 5 practices. We used the Wilcoxon rank‐sum test to test the statistical significance of the relationship between transparency indicators and JIF or received citations. Fisher's exact test with Monte Carlo‐simulated *p*‐values for differences in transparency practices according to publication year, journals, and publishers were performed. Detailed information, deviations from protocol, data, and code are available as Supplementary Material on the OSF repository (osf.io/4eq8u).

## RESULTS

The total number of articles (open access and non‐open access) was 329,784; the full texts of 10,659 (3.2%) were accessible via the EPMC (open access), and, of those, only three were published before 2000, while 1872 (17.6%) were published in 2021 (Figure S1).

The retrieved articles were published in a total of 78 dental journals. When stratified according to article frequency, the five dental journals with the largest number of articles were *BMC Oral Health* (*n* = 2503), *Journal of Applied Oral Science* (*n* = 1440), *Medicina Oral, Patología Oral y Cirugía Bucal* (*n* = 1174), *Head & Face Medicine* (*n* = 572), and *Dental Press Journal of Orthodontics* (*n* = 530). The mean and median number of citations of these articles were 5.7 (SD = 15.0) and 2 (IQR = 7), respectively. The most cited article, with 812 citations, was published in the *International Journal of Oral Science* [18].

**FIGURE 1:**
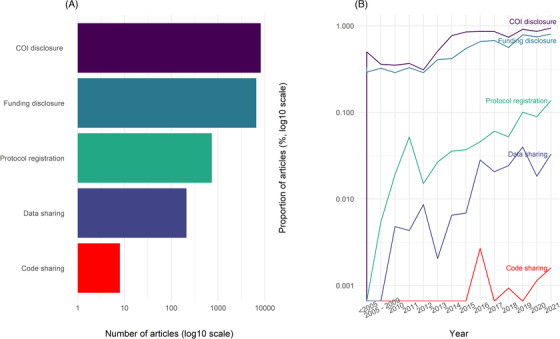
(A) Number of articles adhering to each transparency practice. (B) Proportion of articles adhering to transparency practices over time. COI, conflict of interest.

More than three quarters (76.7%, 95% CI: 75.9–77.5; *n* = 8173) of the articles had a COI disclosure (Figure 1A). Fewer than two‐thirds (61.5%, 95% CI: 60.6–62.4; *n* = 6558) of the articles had a funding disclosure. Seven percent (6.9%, 95% CI: 6.4–7.4; *n* = 737) of the articles had a registered protocol. Two percent of articles (2.0%, 95% CI: 1.7–2.3; *n* = 212) had shared their data. One in a thousand articles (0.1%, 95% CI: 0.04–0.2; *n* = 8) had shared their code.

We observed an increase over time in the proportion of articles with COI disclosure, funding disclosure, and protocol registration, with the proportions highest in 2021 (Figure 1B). One sixth (15.9%, *n* = 1697) of the articles did not adhere to any of the five transparency practices, 28.6% (*n* = 3048) adhered to one, 48.2% (*n* = 5134) adhered to two, and 7.0% (*n* = 748) adhered to three. Less than 1% (0.3%, *n* = 32) of the articles showed adherence to four transparency practices, and none used all five.

Apart from code sharing, we observed journal‐ and publisher‐related differences in transparency practices (Tables 1 and 2, and Supplementary Material at osf.io/4eq8u). Articles in which transparency practices were adhered to were published in journals that had a slightly higher median JIF but fewer citations than those which did not adhere to transparency practices (Table 3). Reviews and interventional studies showed higher adherence to protocol registration (Table 4). The lowest adherence to the five transparency practices was found in articles other than reviews and interventional, observational, and laboratory studies.

**TABLE 1:** Proportion of articles in which adherence to transparency practices was found, stratified according to the five dental journals with the largest number of articles in the sample and the five journals with the highest impact factor

**Adherence to transparency practices**			**Transparency practices**				
The five dental journals in which the largest number of articles were published	***N***	**Open access** **(%)**	**COI disclosure** **(%)**	**Funding disclosure** **(%)**	**Protocol registration** **(%)**	**Data sharing** **(%)**	**Code sharing** **(%)**
*BMC Oral Health*	2503	100	99	89	13	4.8	0.2
*J Appl Oral Sci*	1440	87	21	46	2.2	0.5	0
*Med Oral Patol Oral Cir Bucal*	1174	54	74	42	3.7	0.4	0
*Dental Press J Orthod*	530	81	95	16	2.3	0.4	0
*Head Face Med*	572	100	94	49	4.0	0.3	0
All other journals	4440	1.4	78	63	6.8	1.7	< 0.1

The five journals with the highest impact factor							
*J Clin Periodontol*	79	1.4	92	89	27	6.3	0
*Periodontol*	30	2.7	13	57	0	3.3	0
*J Perodontol*	20	0.2	90	85	15	0	0
*Int J Oral Sci*	447	100	35	66	0.2	1.8	0
*J Dent Res*	73	0.5	100	100	4.1	4.1	0
All other journals	8081	2.7	78	63	7.9	2.1	<0.1
*p‐value*		<0.001	<0.001	<0.001	<0.001	<0.001	0.62

Abbreviation: COI, conflict of interest.

*p*‐Values were calculated using Fisher's Exact Test for Count Data with simulated *p*‐value (based on 2000 replicates).

Open access: proportion of retrieved open articles from all articles published by the journal. Only 8730 articles were stratified accodring to journal impact factor as 1929 articles were published in journals without journal impact factor.

**TABLE 2:** Proportion of articles in which adherence to transparency practices was found, stratified according to the six most common publishers of journals in the sample

	Journal publisher							
Variable	Dental Press	Faculdade de Odontologia de Bauru	Wiley	Medicina Oral, Patologia Oral y Cirugia Bucal	Springer Nature	Wolters Kluwer	All other publishers	*p‐value*
*N*	530	1440	1064	1174	4274	495	1291	
Open access (%)	82	87	1.9	54	19	11	1.0	<0.001
Transparency practice								
COI disclosure (%)	95	21	81	74	95	61	68	<0.001
Funding disclosure (%)	16	46	66	42	75	61	63	<0.001
Protocol registration (%)	2.3	2.2	9.4	3.7	11	0.8	3.1	<0.001
Data sharing (%)	0.4	0.5	3.5	0.4	3.2	0	1.3	<0.001
Code sharing (%)	0	0	<0.1	0	0.2	0	0	0.70

Abbreviation: COI, conflict of interest.

*p*‐Values were calculated using Fisher's Exact Test for Count Data with a simulated *p*‐value (based on 2000 replicates). Only 10268 articles were included in this part of study as 391 articles were published in journals for which no publisher information was available.

Open access: proportion of retrieved open articles from all articles published by the publisher.

**TABLE 3:** Associations of transparency practices with citations of article and journal impact factor

	**Citations of article**			**Journal impact factor**		
	Median (IQR)			Median (IQR)		
**Transparency practice**	Adhered to	Did not adhere to	*p‐value*	Adhered to	Did not adhere to	*p‐value*
COI disclosure	2 (5)	5 (9)	<0.001	2.8 (0.6)	2.7 (0.1)	<0.001
Funding disclosure	2 (5)	3 (7)	<0.001	2.8 (0.1)	2.7 (0.7)	<0.001
Protocol registration	1 (4)	2 (7)	<0.001	2.8 (0.0)	2.7 (0.6)	<0.001
Data sharing	2 (4)	2 (7)	0.01	2.8 (0.0)	2.8 (0.5)	<0.001
Code sharing	1.5 (3)	2 (7)	0.70	2.8 (0.0)	2.8 (0.5)	0.08

Abbreviations: COI, conflict of interest; IQR, interquartile range.

*p*‐Values were calculated using the Wilcoxon rank sum test. Only 8730 articles were stratified accodring to journal impact factor as 1929 articles were published in journals without journal impact factor.

**TABLE 4:** Proportion of articles in which adherence to transparency practices was found stratified according to the type of study

	**Type of Study**					
**Variable**	**Interventional**	**Laboratory**	**Observational**	**Review**	**Other**	***p‐value***
*N*	1252	1885	4530	1303	1689	
Transparency practice						
COI disclosure (%)	80	68	84	80	64	<0.001
Funding disclosure (%)	67	69	65	57	43	<0.001
Protocol registration (%)	27	1.1	4.3	13	0.9	<0.001
Data sharing (%)	1.8	1.5	2.6	2.1	1.0	0.001
Code sharing (%)	0	<0.1	<0.1	0.3	0	0.06

Abbreviation: COI, conflict of interest.

*p*‐Values were calculated using Pearson's chi‐squared test or Fisher's Exact Test for Count Data (based on 2000 replicates).

In the validation sample, one of the articles was an abstract without any full text. Hence, our validation sample consisted of 49 articles. Of these 49 articles, nine (3.6%) discrepancies between automatic tool and manual checking were found: two for open data, four for COI disclosure, one for funding disclosure, and two for registration (see Supplementary Material at osf.io/4eq8u).

## DISCUSSION

Our investigation showed major improvements in COI disclosure, funding disclosure, protocol registration, and data sharing in dental research since 2000. However, still, in 2021, there were major deficiencies, particularly in protocol registration and sharing of data or codes—most articles adhered to only one or two transparency practices. Differences in JIF based on the adherence of articles to transparency indicators were modest. Articles demonstrating adherence to transparency practices had lower citation counts, which may be related to publication more recently than those articles for which adherence to transparency practices was not identified. We also detected study type‐, journal‐, and publisher‐related differences in transparency practices.

Our findings were generally consistent with those of Pandis et al. [5], who reported that adherence to good scientific practices, such as the five transparency practices investigated here, increased in dental research over time. However, we found much higher adherence to COI disclosure (77%) than Pandis et al. [5] (8%–39%). Otherwise, our findings on the prevalence of funding disclosure, protocol registration, and data sharing were in line with the rough estimates from small and very heterogeneous primary studies [5]. For instance, many of the primary studies were restricted to a few leading journals or to some subfield of dentistry [19, 20, 21]. To our knowledge, code sharing has not been investigated in detail in dental research before and, unfortunately, remains rare. We also showed that rarely do dental articles adhere to many transparency practices simultaneously. Thus, there were deficiencies in some of the five aspects of transparency in almost all articles. However, compared with coronavirus disease 2019 (COVID‐19)‐related dental research analyzed using the same methods [22], our findings showed higher adherence to all transparency practices. For instance, much higher adherence to COI disclosure was observed in all dental research (86%–95%, Supplementary Material at osf.io/4eq8u) than in COVID‐19‐related dental research (74%) during the period 2020–2021 [22].

A comparison with almost three million biomedical research articles analyzed using the same automated tools as the present study showed that COI disclosures were included more often in dental research articles than in biomedical articles in 2020 [4]. There was no difference in the prevalence of funding disclosure between dental and biomedical research articles. However, data sharing (2.0%) and code sharing (0.1%) were less common in dental articles than in biomedical research articles (8.9% and 1.2%, respectively) [4]. Protocol registration, conversely, was more common in the dental articles (6.9%) than in the biomedical research articles (2.6%) [4].The proportion of dental articles available from the EPMC (3.2%) is smaller than that of all biomedical (PubMed) articles (24%, Supplementary Material at osf.io/4eq8u), and information is unavailable regarding whether these samples are equally representative of all (open and non‐open access) dental and biomedical articles. However, it seems that the proportion of open access dental articles increases considerably over time [23]. It is also possible that differences in the research curriculum or journal styles could explain these discrepancies, and this should be clarified in future studies.

Many authors and institutions have highlighted the benefits of transparent science, namely to increase reliability, credibility, and reproducibility of science, and reduce research waste [2, 24, 25]. All funders, publishers, societies, institutions, editors, reviewers, and authors have the responsibility to improve scientific practices, including transparency [2]. For instance, stakeholders could radically increase the incentives for implementing good scientific practices by using the Registered Report publishing format, where peer‐review for publication and funding could be conducted simultaneously in the design phase before data collection, in addition to “normal” peer‐review when reporting study findings [26, 27]. This would improve research transparency in light of current open science and transparency standards in many journals across fields [28, 29, 30]. Our positive findings of increased inclusion of COI disclosure in recent years are likely the result of long and determined efforts, indicating that major developments in transparency are possible in the long term [31]. However, registration of interventional studies, such as randomized controlled trials, or systematic reviews can be significantly improved [7, 32–34]. For instance, stricter adherence to journal policies is needed from authors but also from journals themselves [7]. On the other hand, one can say that wider adherence to transparent practices would only modestly improve the current state of science because no amount of transparency can overcome the systematic bias stemming from publication and impact‐driven “publish or perish” science [35, 36, 37]. For instance, in addition to advancing preregistration and sharing data and code, evidently more detailed, complete, and comprehensible reporting of how research was carried out is needed to improve the replicability and credibility of research [3].

There are some limitations and features of our investigation that have to be noted when interpreting our findings. One is that we did not analyze the content or appropriateness of COI or funding statements or protocol registration. Of concern, earlier studies have implied that actually few dental studies report any COI or sponsorship from for‐profit sources [38] and that most registered dental studies are registered retrospectively [7]. Second, the algorithms captured only free data and code sharing, which is problematic if one has not used any data or performed any statistical analyses (as there is no data or code to share). It is also evident that not all research data can be made readily available, for instance because of privacy issues or requirements of data owners. Sometimes data are made available on reasonable request from the authors. However, a recent study indicated problems related to sharing data on reasonable request: only 7% of authors who declared that they would share data on reasonable request actually did so when their data were requested [39].

The study sample was restricted to open access articles in the EPMC database and thus the sample may not represent all research articles published in dental journals. However, investigation of biomedical research articles has shown that, in terms of these five transparency indicators, there are only small differences between articles accessible and inaccessible via the EPMC [40, 41]. On the other hand, there are some notable differences between open access and non‐open access articles: open access articles are more frequently published by men, people employed at prestigious institutions, those with greater federal research funding, and those in more advanced career stages [42]. It is also possible that despite our attempts to exclude all non‐research articles, some proportion of articles had no data or code to share or no need to register a protocol (such as commentaries). In addition, as our validation analyses showed, some articles included only abstracts and lacked the information required for a complete evaluation. However, the validation sample showed that the methods applied are approriate for investigation of transparency in dental research articles. Finally, one should note that because we investigated only information in published research articles, we do not know whether relevant information has been delivered during the submission and peer review process. It is hoped that the absence of funding or COI statements in published articles is simply because of a decision not to publish such information; for instance, as a result of journal style not to publish funding statements if authors report they received no external funding. Investigations of journal submission and peer review policies, and adherence to them, would provide valuable knowledge about what the dental journals demand in terms of transparency and why some articles lack funding disclosure, COI disclosure or protocol registration statements.

We showed that adherence to transparency practices in studies published in dental journals available via the EPMC has increased between 2000 and 2021, but data and code sharing remained rare, even in 2021. Coordinated efforts involving all stakeholders are needed to provide further improvements in the transparency of dental science.

## AUTHOR CONTRIBUTIONS

**Conceptualization**: Eero Raittio, Ahmad Sofi‐Mahmudi, Sergio Uribe; **Methodology**: Ahmad Sofi‐Mahmudi, Eero Raittio; **Software**: Ahmad Sofi‐Mahmudi, Eero Raittio; **Validation**: Ahmad Sofi‐Mahmudi, Eero Raittio; **Formal analysis**: Eero Raittio, Ahmad Sofi‐Mahmudi; **Investigation**: Eero Raittio, Ahmad Sofi‐Mahmudi; **Resources**: Eero Raittio; **Data Curation;** Ahmad Sofi‐Mahmudi, Eero Raittio; **Writing – original draft preparation**: Eero Raittio, Ahmad Sofi‐Mahmudi; **Writing – review and editing**: Sergio Uribe, Ahmad Sofi‐Mahmudi; **Visualization**: Ahmad Sofi‐Mahmudi, Sergio Uribe, Eero Raittio; **Supervision**: Sergio Uribe; **Project administration**: Ahmad Sofi‐Mahmudi, Eero Raittio; **Funding acquisition**: Eero Raittio, Ahmad Sofi‐Mahmudi.

## CONFLICTS OF INTEREST

The authors have no conflicts of interest to disclose.

## Supporting information

SUPPORTING INFORMATION

## References

[1] Schwendicke F , Marazita ML , Jakubovics NS , Krois J . Big data and complex data analytics: breaking peer review? J Dent Res. 2022;101:369–70. 35048725 10.1177/00220345211070983 PMC8935526

[2] Munafò MR , Nosek BA , Bishop DVM , Button KS , Chambers CD , Percie du Sert N , et al. A manifesto for reproducible science. Nat Hum Behav. 2017;1:0021. 10.1038/s41562-016-0021 33954258 PMC7610724

[3] Errington TM , Denis A , Perfito N , Iorns E , Nosek BA . Challenges for assessing replicability in preclinical cancer biology. Elife. 2021;10:e67995. 10.7554/eLife.67995 34874008 PMC8651289

[4] Serghiou S , Contopoulos‐Ioannidis DG , Boyack KW , Riedel N , Wallach JD , Ioannidis JPA . Assessment of transparency indicators across the biomedical literature: how open is open? PLOS Biol. 2021;19:e3001107. 10.1371/journal.pbio.3001107 33647013 PMC7951980

[5] Pandis N , Fleming PS , Katsaros C , Ioannidis JPA . Dental research waste in design, analysis, and reporting: a scoping review. J Dent Res. 2021;100:245–52. 33054504 10.1177/0022034520962751

[6] Faggion CM Jr Listl S , Smits KPJ . Meta‐research publications in dentistry: a review. Eur J Oral Sci. 2021;129:e12748. 10.1111/eos.12748 33533130

[7] Smaïl‐Faugeron V , Fron‐Chabouis H , Durieux P . Clinical trial registration in oral health journals. J Dent Res. 2015;94(Suppl 3):8S–13S. 25274753 10.1177/0022034514552492 PMC4541084

[8] Almaqrami BS , Hua F , Liu Y , He H . Research waste‐related editorial policies of leading dental journals: situation 2018. Oral Dis. 2020;26:696–706. 31845484 10.1111/odi.13257

[9] Ioannidis JPA , Greenland S , Hlatky MA , Khoury MJ , Macleod MR , Moher D , et al. Increasing value and reducing waste in research design, conduct, and analysis. Lancet. 2014;383:166–75. 24411645 10.1016/S0140-6736(13)62227-8 PMC4697939

[10] Nasser M , Clarke M , Chalmers I , Brurberg KG , Nykvist H , Lund H , et al. What are funders doing to minimise waste in research? Lancet. 2017;389:1006–7. 28290987 10.1016/S0140-6736(17)30657-8

[11] Curry S , de Rijcke S , Hatch A , Pillay DG , van der Weijden I , Wilsdon J . The changing role of funders in responsible research assessment: progress, obstacles and the way ahead. Research on Research Institute; 2020. Available from: https://rori.figshare.com/articles/report/The_changing_role_of_funders_in_responsible_research_assessment_progress_obstacles_and_the_way_ahead/13227914/1 Accessed 13 Sep 2020.

[12] Serghiou S . metareadr. Downloads data often needed for meta‐research. 2022. https://github.com/serghiou/metareadr

[13] Avau B , Van Remoortel H , De Buck E . Translation and validation of PubMed and Embase search filters for identification of systematic reviews, intervention studies, and observational studies in the field of first aid. J Med Libr Assoc. 2021;109:599–608. 34858089 10.5195/jmla.2021.1219 PMC8608173

[14] R Core Team . R: a language and environment for statistical computing. Vienna: R Foundation for Statistical Computing; 2021. https://www.R‐project.org/

[15] Jahn N . europepmc: R Interface to the Europe PubMed Central RESTful Web Service. 2021. https://CRAN.R‐project.org/package=europepmc

[16] Wickham H . ggplot2: elegant graphics for data analysis. 2nd ed. Berlin: Springer; 2016. https://ggplot2.tidyverse.org

[17] Stevenson M , Sergeant E . epiR: tools for the analysis of epidemiological data. 2022. https://CRAN.R‐project.org/package=epiR

[18] Xu H , Zhong L , Deng J , Peng J , Dan H , Zeng X , et al. High expression of ACE2 receptor of 2019‐nCoV on the epithelial cells of oral mucosa. Int J Oral Sci. 2020;12:8. 10.1038/s41368-020-0074-x 32094336 PMC7039956

[19] Alamri HM , Alharbi F . Quality assessment of randomized clinical trials reporting in endodontic journals: an observational study from 2012 to 2017. J Endod. 2018;44:1246–50. 30053935 10.1016/j.joen.2018.05.011

[20] Kiriakou J , Pandis N , Fleming PS , Madianos P , Polychronopoulou A . Reporting quality of systematic review abstracts in leading oral implantology journals. J Dent. 2013;41:1181–7. 24075952 10.1016/j.jdent.2013.09.006

[21] Papageorgiou SN , Antonoglou GN , Martin C , Eliades T . Methods, transparency and reporting of clinical trials in orthodontics and periodontics. J Orthod. 2019;46:101–9. 31066609 10.1177/1465312519842315

[22] Sofi‐Mahmudi A , Raittio E . Transparency of COVID‐19‐related research in dental journals. Front Oral Health. 2022;3:871033. 10.3389/froh.2022.871033 35464778 PMC9019132

[23] Yu X , Meng Z , Qin D , Shen C , Hua F . The long‐term influence of Open Access on the scientific and social impact of dental journal articles: an updated analysis. J Dent. 2022;119:104067. 10.1016/j.jdent.2022.104067 35167896

[24] Allen C , Mehler DMA . Open science challenges, benefits and tips in early career and beyond. PLOS Biol. 2019;17:e3000246. 10.1371/journal.pbio.3000246 31042704 PMC6513108

[25] Besançon L , Peiffer‐Smadja N , Segalas C , Jiang H , Masuzzo P , Smout C , et al. Open science saves lives: lessons from the COVID‐19 pandemic. BMC Med Res Methodol. 2021;21:117. 10.1186/s12874-021-01304-y 34090351 PMC8179078

[26] Nosek BA , Lakens D . Registered Reports. Soc Psychol. 2014;45:137–41.

[27] Chambers C . What's next for registered reports? Nature. 2019;573:187–9. 31506624 10.1038/d41586-019-02674-6

[28] Nosek BA , Alter G , Banks GC , Borsboom D , Bowman SD , Breckler SJ , et al. Promoting an open research culture. Science. 2015;348:1422–5. 26113702 10.1126/science.aab2374 PMC4550299

[29] Cashin AG , Bagg MK , Richards GC , Toomey E , McAuley JH , Lee H . Limited engagement with transparent and open science standards in the policies of pain journals: a cross‐sectional evaluation. BMJ Evid‐Based Med. 2020;26:313–9. 10.1136/bmjebm-2019-111296 31980469

[30] Hansford HJ , Cashin AG , Wewege MA , Ferraro MC , McAuley JH , Jones MD . Open and transparent sports science research: the role of journals to move the field forward. Knee Surg Sports Traumatol Arthrosc. 2022;30:3599–601. 35092443 10.1007/s00167-022-06893-9

[31] Blum JA . Requirements and definitions in conflict of interest policies of medical journals. JAMA. 2009;302:2230. 19934424 10.1001/jama.2009.1669

[32] Weber WEJ , Merino JG , Loder E . Trial registration 10 years on. BMJ. 2015;351:h3572. 10.1136/bmj.h3572 26149708

[33] Straus S , Moher D . Registering systematic reviews. CMAJ Can Med Assoc J J Assoc Medicale Can. 2009/07/20 ed. 2010;182:13–4. 10.1503/cmaj.081849 PMC2802597 19620270

[34] Cenci MS , Franco MC , Raggio DP , Moher D , Pereira‐Cenci T . Transparency in clinical trials: adding value to paediatric dental research. Int J Paediatr Dent. 2020;31(Suppl 1):4–13. 33314319 10.1111/ipd.12769

[35] Fanelli D . Do pressures to publish increase scientists’ bias? An empirical support from us states data. PLoS One. 2010;5:e10271. 10.1371/journal.pone.0010271 20422014 PMC2858206

[36] Altman DG . The scandal of poor medical research. BMJ. 1994;308:283–4. 8124111 10.1136/bmj.308.6924.283 PMC2539276

[37] Gelman A . Ethics and statistics: honesty and transparency are not enough. CHANCE. 2017;30:37–9.

[38] Faggion CM , Pandis N , Cardoso GC , Rodolfo B , Morel LL , Moraes RR . Reporting of conflict of interest and sponsorship in dental journals. J Dent. 2020;102:103452. 10.1016/j.jdent.2020.103452 32805358

[39] Gabelica M , Bojčić R , Puljak L . Many researchers were not compliant with their published data sharing statement: a mixed‐methods study. J Clin Epidemiol. 2022;150:33–41. 35654271 10.1016/j.jclinepi.2022.05.019

[40] Wallach JD , Boyack KW , Ioannidis JPA . Reproducible research practices, transparency, and open access data in the biomedical literature, 2015–2017. PLOS Biol. 2018;16:e2006930. 10.1371/journal.pbio.2006930 30457984 PMC6245499

[41] Uribe SE , Sofi‐Mahmudi A , Raittio E , Maldupa I , Vilne B . Dental research data availability and quality according to the FAIR principles. J Dent Res. 2022;101:1307–13. 35656591 10.1177/00220345221101321 PMC9516597

[42] Olejniczak AJ , Wilson MJ . Who's writing open access (OA) articles? Characteristics of OA authors at Ph.D.‐granting institutions in the United States. Quant Sci Stud. 2020;1(4):1429–50.

