# Supplementary material for: Research transparency in dental research: A programmatic analysis
Source: Eur J Oral Sci. 2022 Dec 8;131(1):e12908. doi: 10.1111/eos.12908 (PMC10108147; doi:10.1111/eos.12908)

SUPPORTING INFORMATION

Research transparency in dental research: a programmatic analysis

RAITTIO E, SOFI-MAHMUDI A, URIBE SE

University of Eastern Finland, Kuopio, Finland
Aarhus University, Aarhus, Denmark
Kurdistan University of Medical Sciences, Seqiz, Kurdistan
McMaster University, Hamilton, ON, Canada
Riga Stradins University, Riga, Latvia
Universidad Austral de Chile, Valdivia, Chile
Riga Technical University, Riga, Latvia

Figure S1. Number of articles in the sample by year of publication.

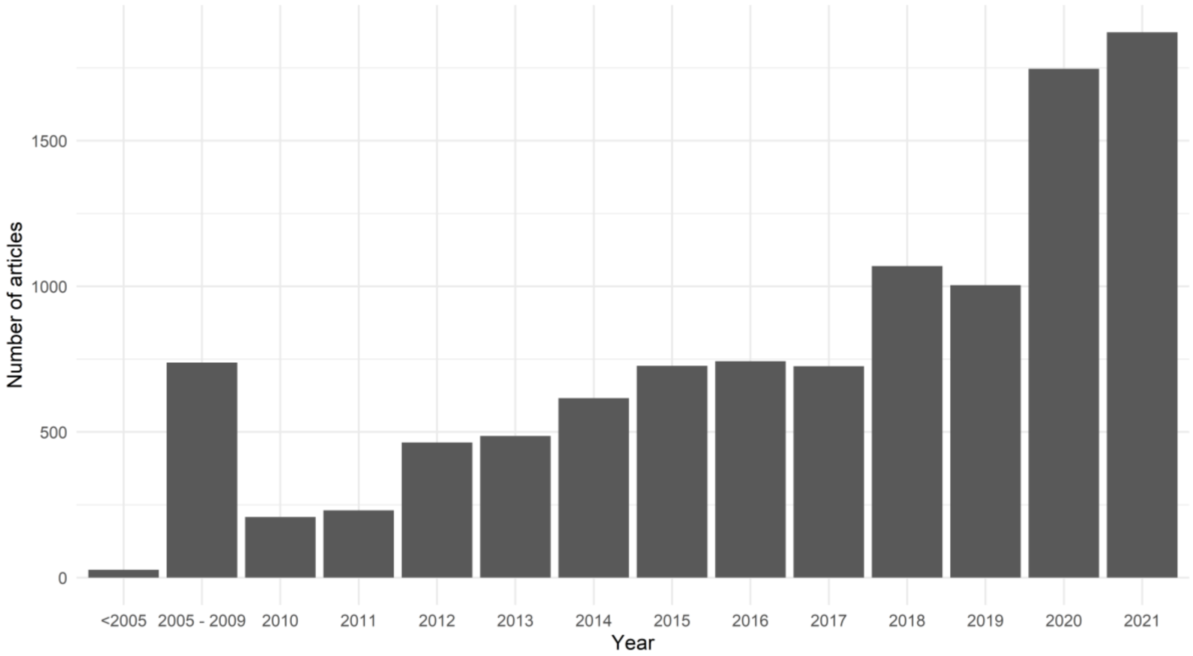

Supplement: Supplementary file 1 — SUPPORTING INFORMATION [file EOS-131-0-s001.pdf]
